# Dynamics of tongue microbial communities with single-nucleotide resolution using oligotyping

**DOI:** 10.3389/fmicb.2014.00568

**Published:** 2014-11-07

**Authors:** Jessica L. Mark Welch, Daniel R. Utter, Blair J. Rossetti, David B. Mark Welch, A. Murat Eren, Gary G. Borisy

**Affiliations:** ^1^Josephine Bay Paul Center for Comparative Molecular Biology and Evolution, Marine Biological LaboratoryWoods Hole, MA, USA; ^2^Department of Microbiology, The Forsyth InstituteCambridge, MA, USA

**Keywords:** human microbiome, oral microbiota, 16S ribosomal RNA, *Haemophilus*, *Neisseria*, *Streptococcus*, *Veillonella*

## Abstract

The human mouth is an excellent system to study the dynamics of microbial communities and their interactions with their host. We employed oligotyping to analyze, with single-nucleotide resolution, oral microbial 16S ribosomal RNA (rRNA) gene sequence data from a time course sampled from the tongue of two individuals, and we interpret our results in the context of oligotypes that we previously identified in the oral data from the Human Microbiome Project. Our previous work established that many of these oligotypes had dramatically different distributions between individuals and across oral habitats, suggesting that they represented functionally different organisms. Here we demonstrate the presence of a consistent tongue microbiome but with rapidly fluctuating proportions of the characteristic taxa. In some cases closely related oligotypes representing strains or variants within a single species displayed fluctuating relative abundances over time, while in other cases an initially dominant oligotype was replaced by another oligotype of the same species. We use this high temporal and taxonomic level of resolution to detect correlated changes in oligotype abundance that could indicate which taxa likely interact synergistically or occupy similar habitats, and which likely interact antagonistically or prefer distinct habitats. For example, we found a strong correlation in abundance over time between two oligotypes from different families of Gamma Proteobacteria, suggesting a close functional or ecological relationship between them. In summary, the tongue is colonized by a microbial community of moderate complexity whose proportional abundance fluctuates widely on time scales of days. The drivers and functional consequences of these community dynamics are not known, but we expect they will prove tractable to future, targeted studies employing taxonomically resolved analysis of high-throughput sequencing data sampled at appropriate temporal intervals and spatial scales.

## Introduction

Understanding microbial community dynamics requires knowledge of the time scale over which microbial communities adapt and change. Studies using rRNA gene-based approaches to investigate microbial communities sampled at intervals of weeks to months found that these communities correlated to environmental conditions (Fuhrman et al., 2006; Dethlefsen et al., 2008; Gilbert et al., 2012; Chow et al., 2013). Indications that changes of interest may occur over shorter time scales led to studies that sampled at daily intervals in a marine system and in the human microbiome (Dethlefsen and Relman, 2011; Caporaso et al., 2011; Koenig et al., 2011; Gajer et al., 2012; Martínez et al., 2013; Needham et al., 2013; David et al., 2014). These studies established that microbial communities are resilient, with episodic shifts in community composition followed by reversion to previous states. Remarkably, within that overall stability, dramatic fluctuations in community composition could occur on time scales of the order of days.

Our understanding of microbial community dynamics at the species level has heretofore been hindered by the use of analysis methods that cluster sequences into operational taxonomic units (OTUs) based on arbitrary similarity thresholds. Such methods have the twin drawbacks that they generate heterogeneous groupings of limited biological relevance and that they do not make full use of available sequence information that would allow finer taxonomic resolution. Many described microbial species differ by only 1 or 2% in rRNA gene sequence, yet standard analysis methods lump them together by clustering sequences that are more than 97% identical. A recently developed computational method called oligotyping (Eren et al., 2013) removes this hindrance. Oligotyping uses a calculation of Shannon entropy to identify nucleotide positions of high variation (i.e., high information content) in a dataset, and employs only these positions to partition the sequence dataset into groups called oligotypes. It exploits all available informative data, reduces the effect of noise, and generates homogeneous groupings in the sense that nearly every read assigned to an oligotype, if classified individually by BLAST, would have the same taxonomic annotation (Eren et al., 2014). Oligotyping allows the analysis of high-throughput sequencing datasets with single-nucleotide resolution. A different approach that also achieves single-nucleotide resolution has recently been reported (Tikhonov et al., 2014).

Application of oligotyping to the human oral microbiota presents an opportunity to analyze a tractable microbial community with a level of taxonomic resolution that permits differentiation among important species and, in favorable cases, analysis of within-species dynamics. The human mouth is an excellent test bed for microbiome analysis for several reasons: it is home to a well-studied microbial community for which a highly curated Human Oral Microbiome Database (HOMD) (www.homd.org) has been established (Dewhirst et al., 2010); a high proportion of the human oral microbiota have been cultured (65%); fully sequenced genomes are available for many (50%) of the oral microbiota; and, importantly, a foundation for defining the healthy human oral microbiome has been laid by the Human Microbiome Project (HMP) (http://commonfund.nih.gov/hmp/index.aspx) which sampled nine oral sites from over 200 healthy individuals and generated millions of sequences (Human Microbiome Project Consortium, 2012).

The oral microbiota may be deconstructed into overlapping but distinct communities. For example, the human tongue is the substrate for an abundant microbiota different in composition from the microbiota on the teeth and on the mucosal surfaces of the mouth, as first indicated by distinctive distribution of a few taxa in DNA hybridization and early sequencing studies (Mager et al., 2003; Aas et al., 2005; Socransky and Haffajee, 2005; Zaura et al., 2009). Analysis of the HMP data confirmed the finding of broad differences in the microbiome of the tongue dorsum as compared to plaque and to the surfaces of the gums, cheek and hard palate (Segata et al., 2012).

The application of oligotyping to the HMP data for the oral microbiome (Eren et al., 2014), in combination with habitat analysis of oligotype distribution across nine oral sites, identified a level of ecological and functional biodiversity in the oral microbiome not previously recognized. We identified oral site-specialists, established correlations between sites within individual mouths, and revealed predominance of certain oligotypes within individuals that would not have been seen with OTU clustering. Some oligotypes differing by a few nucleotides or even as little as a single nucleotide showed strikingly different distributions across oral sites or among individuals, suggesting that even single-nucleotide differences in the 16S rRNA gene can act as markers for underlying, biologically significant differences elsewhere in the genome.

The HMP data provided an invaluable baseline for assessing variation in the microbiome across a range of individuals whose health status was carefully documented. However, this baseline represents a single “snapshot” in time from each of the sampled individuals, meaning that the significance of some distributional patterns of oligotypes remained unclear. Some very closely related oligotypes, for example representing different species of *Streptococcus*, were detected in the tongue of every individual, but in widely different proportions in different individuals; were these proportions a stable characteristic of an individual's microbiota or did they change over time and over what time scales? Other closely related oligotypes apparently represented different strains within a single species. For example, in the *Neisseria flavescens/subflava* group, one or another of these oligotypes would dominate the tongue community in an individual, making up 90% or more of the reads of that taxon. Is one oligotype stably dominant in each individual, or does the dominance relationship fluctuate?

A time-resolved high-throughput sequencing dataset from the tongue of two individuals (Caporaso et al., 2011) provided an ideal opportunity to test the stability of these distributions over time as well as to generate a more precise and unified description of the characteristic microbiota of the tongue. We carried out oligotyping on this dataset and compared the resulting oligotypes to those detected in HMP data. Oligotyping, similar to other *de novo* partitioning approaches, creates units that are dataset-specific and not inherently comparable across datasets. We overcame this limitation by making taxonomic assessments for each oligotype by reference to the HOMD. This association of oligotypes from separate datasets allowed us to apply the insights gained from a large time-series study of two individuals to the analysis of a large cross-sectional study with many individuals. It also provided resolution sufficient to discriminate very closely-related taxa, so that for the first time we can describe with species-level or near-species level precision the overall composition and temporal dynamics of the tongue microbial community.

## Methods

### Sample collection

This is a re-analysis of existing sequence data; procedures for informed consent, institutional review board approval, and sample collection and sequencing are described in the original publications (Caporaso et al., 2011; The Human Microbiome Project Consortium, 2012; Aagaard et al., 2013).

### Preparing the sequence data

The study by Caporaso et al. (2011) describes in detail the sample collection, sequencing, and quality filtering of reads used in this study. Briefly, one male and one female adult were sampled approximately daily over 15 months (male) and 6 months (female). The V4 region of the 16S rRNA gene was amplified from tongue samples and amplicons were sequenced using the Illumina HiSeq platform (Illumina, Inc., San Diego, CA, USA). We obtained the quality-filtered data from MG-RAST (http://metagenomics.anl.gov/) using sample accession IDs 4457768.3 through 4459735.3. To eliminate the artificial length variation among reads introduced by the original quality trimming, we re-trimmed each read to 130 nucleotides, and removed the reads that were shorter. For each sample with >20,000 reads we randomly subsampled to 20,000 reads to minimize the sampling bias in our results. The resulting dataset contained 508 samples and a total of 7,538,132 sequencing reads. We used GAST (Huse et al., 2008) to assign taxonomy at the family level to each read in the dataset.

### Oligotyping

We used oligotyping pipeline version 1.0 available from http://oligotyping.org (Eren et al., 2013) on each taxonomic family separately. For each family, we used the auto component command (-c) to select the two nucleotide positions with the highest Shannon entropy, partioning each family into up to eight groups. Groups were further divided by manually adding additional nucleotide positions (using the -C parameter) based on the recalculated Shannon entropy and on the absolute and relative abundance distribution among samples of the unique sequences within a grouping. No more than 5 nucleotide positions were added in a single iteration. The minimum substantive abundance threshold for an oligotype (-M) was set to 500 reads. Upon completion of the oligotyping analysis for each family, we concatenated the resulting observation matrices to generate a single observation matrix reporting counts (i.e. number of reads assigned to each oligotype in each sample). We also converted counts to percent abundances within each sample and used these normalized relative abundances for all analyses except the cross-correlation analysis which was performed on the count data. We assigned taxonomic values to each oligotype by a BLAST search using NCBI executables (--blast-ref-db) against the HOMD RefSeq v.12.0 obtained from www.homd.org. Each oligotype was assigned the taxonomy of the closest match(es) in HOMD except for the one oligotype that had no match within 90% of any sequence in HOMD.

### Cross-correlation and auto-correlation analysis

We carried out cross-correlation analysis using Matlab R2014a (version 8.3) using the counts matrix for each oligotype (the number of reads assigned to each oligotype in each sample) and using the percent matrix (the counts normalized within each sample). Results using Pearson cross-correlation are shown (Matlab function corr); we also carried out the same analysis using Spearman and Kendall with comparable results. Significance (*p*-value) was calculated using the corr function which employs a Student's t distribution for a transformation of the correlation. We used the Bonferroni correction for multiple tests by multiplying significance estimates by 315^2^ ~= 10^5^. Auto-correlation analysis was carried out using the Matlab function xcorr on percent-normalized data for the entire time course for each subject and for subsets of the male time course, and in each case was evaluated over a window of plus or minus 21 days. Potential periodicity of oligotype abundance was analyzed with Fourier transforms using the Matlab functions fft and periodogram. For this analysis, linear interpolation was used to estimate the relative abundance of oligotypes on days without sequencing data.

### Analysis of V3-V5 reads from HMP data for multiple time points

We used the HMP 16S sequence data from the V3-V5 region. Quality filtering and trimming, chimera removal, and taxonomic assignment of reads were previously performed (The Human Microbiome Project Consortium, 2012) using mothur (Schloss et al., 2011) and the reads were uploaded into a MySQL database. From this data we selected subjects from whom two tongue dorsum samples were available with at least 600 reads from each sample. We counted the number of reads assigned to each genus in each sample, and clustered this abundance data using the Morisita-Horn dissimilarity index.

### BLAST searches of microbial genomes

We conducted BLAST searches at HOMD (www.homd.org) using blastn against all oral microbial genomic DNAs annotated by HOMD, and at NCBI (www.ncbi.nlm.nih.gov) using megablast against all completed microbial genomes and against draft genomes of *Haemophilus* and *Neisseriaceae*.

## Results

### Oligotyping results

We used oligotyping to re-analyze time series data sampled from the tongues of two individuals at up to 396 time points (Caporaso et al., 2011). We oligotyped each of the 17 most abundant bacterial families, selecting sets of sequence reads based on their family-level taxonomic assignment using GAST (Huse et al., 2008). These 17 families together represented 99% of reads in the combined tongue data set, and this family-level oligotyping achieved a similarly comprehensive result to the phylum-level oligotyping of HMP data as previously described (Eren et al., 2014) but with lower complexity in the supervision process. The number of nucleotides we used to define oligotypes in the time series data set ranged from 3 (for Actinomycetaceae and Bacillales) to 24 (for Neisseriaceae). We partitioned the data into 315 oligotypes (Table S1) and assigned taxonomic identification to each by BLAST search of the representative sequence for each oligotype against the Human Oral Microbiome Database (HOMD, Dewhirst et al., 2010). Oligotyping of 16S rRNA gene tag sequence data from the tongue dorsum as well as eight other oral sites for 148 individuals sequenced in the V3-V5 region, and 77 individuals sequenced in the V1-V3 region, was previously described (Eren et al., 2014). Results from that study provide the foundation for the current study.

### Phase transition of a microbial community

**Figure 1:**
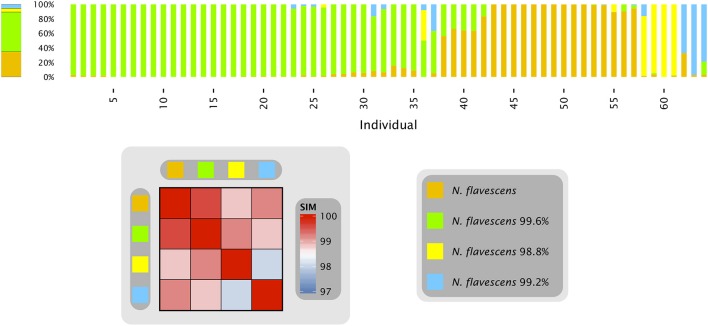
***Neisseria* oligotypes in the tongue**. Human Microbiome Project data from the tongue, sequenced in the V1-V3 region. The four oligotypes with at least 0.5% mean abundance across all sampled individuals are shown with their percent identity to *N. flavescens*, the closest match in HOMD. Colored bars represent the relative abundance of these four oligotypes in samples from an individual tongue; the 64 samples shown are those in which these *Neisseria* oligotypes represented a total of at least 10 reads. The bar on the left represents the relative abundance of each of these four oligotypes averaged across all 64 tongue samples. The heat map shows the similarity of each pair of oligotypes. Data are from Eren et al. (2014).

With the single-nucleotide resolution achievable by oligotyping, strains or variants within a taxon that differ in their rRNA sequence are in principle detectable and their population dynamics open to analysis. We previously found, for example, a case of closely-related oligotypes within the genus *Neisseria*, in which the *Neisseria* population on the tongue of each subject was dramatically different from the mean abundance of the oligotypes across all sampled subjects. Remarkably, the *Neisseria* population on an individual tongue was generally dominated by one or another of these oligotypes (Figure 1 and Eren et al., 2014). To understand the cause of this distributional pattern, it is important to know whether the differences between individuals are stable, or whether populations within individuals change over time.

When only a single sample is analyzed from each of many individuals, as in Figure 1, it is impossible to assess whether populations within an individual are stable or dynamic. Most individuals possessed a population in which a single oligotype made up at least 90% of the *Neisseria* reads, but the oligotype varied from individual to individual, suggesting the possibility of multiple stable states, each dominated by a single *Neisseria* oligotype. However, some individuals had populations lacking a dominant oligotype. Did the more mixed populations represent short-lived transitions between the stable states in individuals who were by chance sampled during the transition? Alternatively, did certain individuals stably maintain a mixed *Neisseria* population?

**Figure 2:**
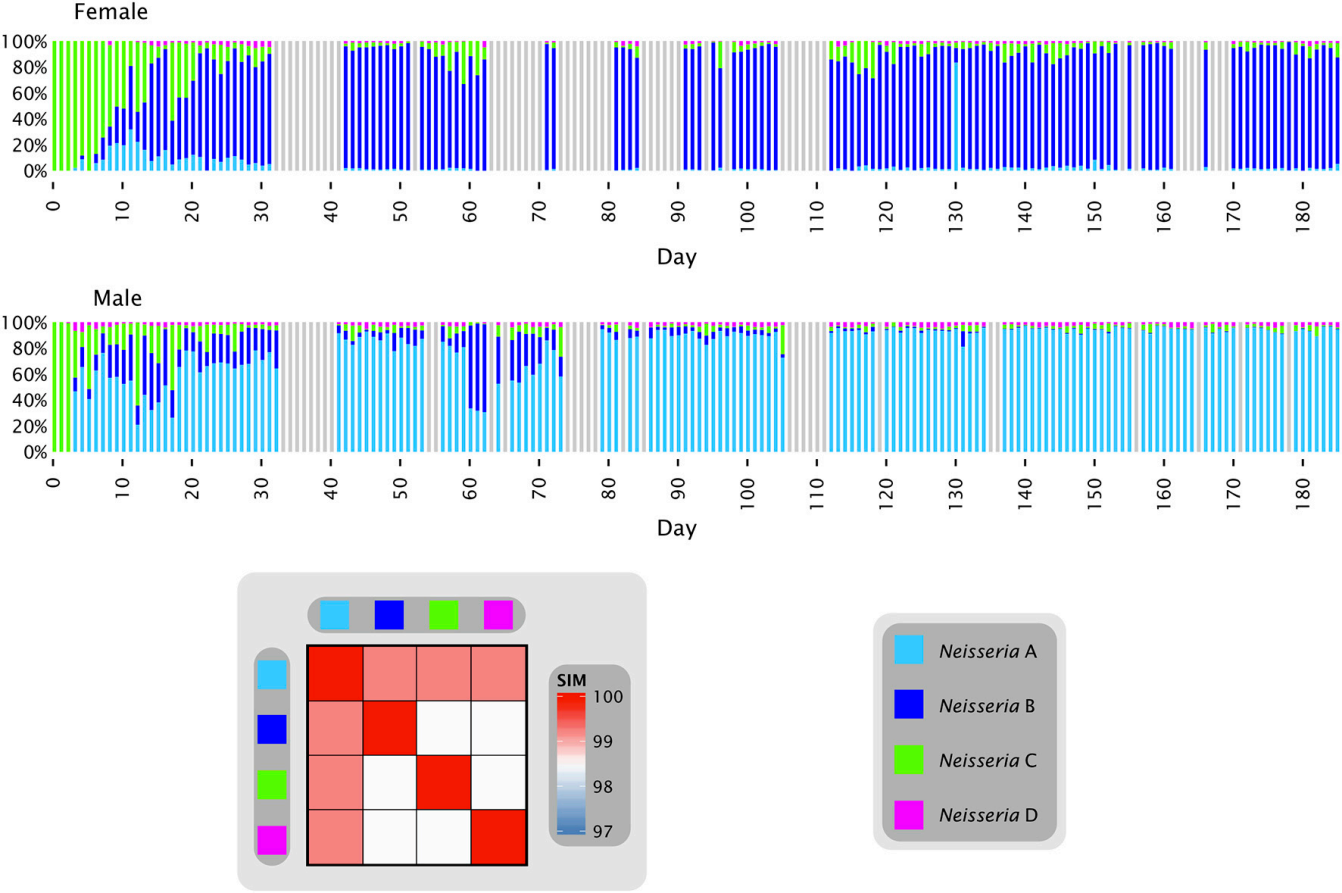
***Neisseria* oligotypes in time series data from the tongue**. Oligotypes with at least 0.5% mean abundance in at least one of the two individuals are shown. Colored bars represent the relative abundance of each of the four oligotypes in a single sample; data shown is for days 0 to 185 and gray bars represent days for which data is unavailable. The heat map shows the similarity of each pair of oligotypes. *Neisseria* A (oligo_001 in Table S1) is 100% identical to the HOMD reference sequence of *N. subflava*; *Neisseria* B (oligo_011) and *Neisseria* D (oligo_024) are each 99.2% identical to the *N. subflava* reference sequence; and *Neisseria* C (oligo_017) is identical to the reference sequences for *N. mucosa, N. flava, N. pharyngis, N. oralis*, and *Neisseria* spp. Human Oral Taxon (HOT) 015 and 018. All are within 98% identity of one another.

Oligotyping of a time series from the tongue of two individuals (Figure 2) answered some of these questions and provided plausible explanations for the observed distributions. Most of the *Neisseria* in both subjects consisted of three major oligotypes, shown in Figure 2 as light blue (*Neisseria* A), dark blue (*Neisseria* B), and green (*Neisseria* C) with a small amount of a fourth oligotype shown as magenta (*Neisseria* D). The *Neisseria* population in both subjects was initially dominated by type C, which was the only *Neisseria* oligotype detectable in the first three samples from the female and two samples from the male. The additional types A and B then became detectable in both individuals, increasing rapidly as a proportion of the total *Neisseria* (Figure 2). In the female, type A was initially the more abundant of these two, but rapidly faded in abundance relative to type B, which became the dominant *Neisseria* in the female after approximately day 35. In the male, by contrast, type B increased and then decreased in relative abundance several times before diminishing in proportion until its abundance was negligible and the population was dominated by type A after approximately day 100. These dynamics display two main characteristics which, taken together, may be termed a phase transition. The major behavior is one of stability. For most of the time, the oligotype distribution within an individual was essentially invariant, irrespective of whether the dominant oligotype in the individual was type A or type B. The second property was of abrupt transition to an alternate oligotype. The time series data showed several instances in which a community initially dominated by one oligotype became transiently mixed and then transitioned to a state where one oligotype was dominant. These properties suggest that the evenly mixed populations of *Neisseria* on the tongue found in some individuals in the HMP data are transient states. Occasional replacement of the dominant oligotype argues against strong founder effects and priority effects for this taxon in the tongue microbiota. Throughout these transitions the fourth oligotype, type D, did not participate in the apparently competitive or exclusionary dynamics of types A and B, but persisted in relatively stable proportion in the community, likely demonstrating a subdivision of functional/ecological roles even among these very closely related taxa.

### Differences among individuals are comparable to fluctuations over time

**Figure 3:**
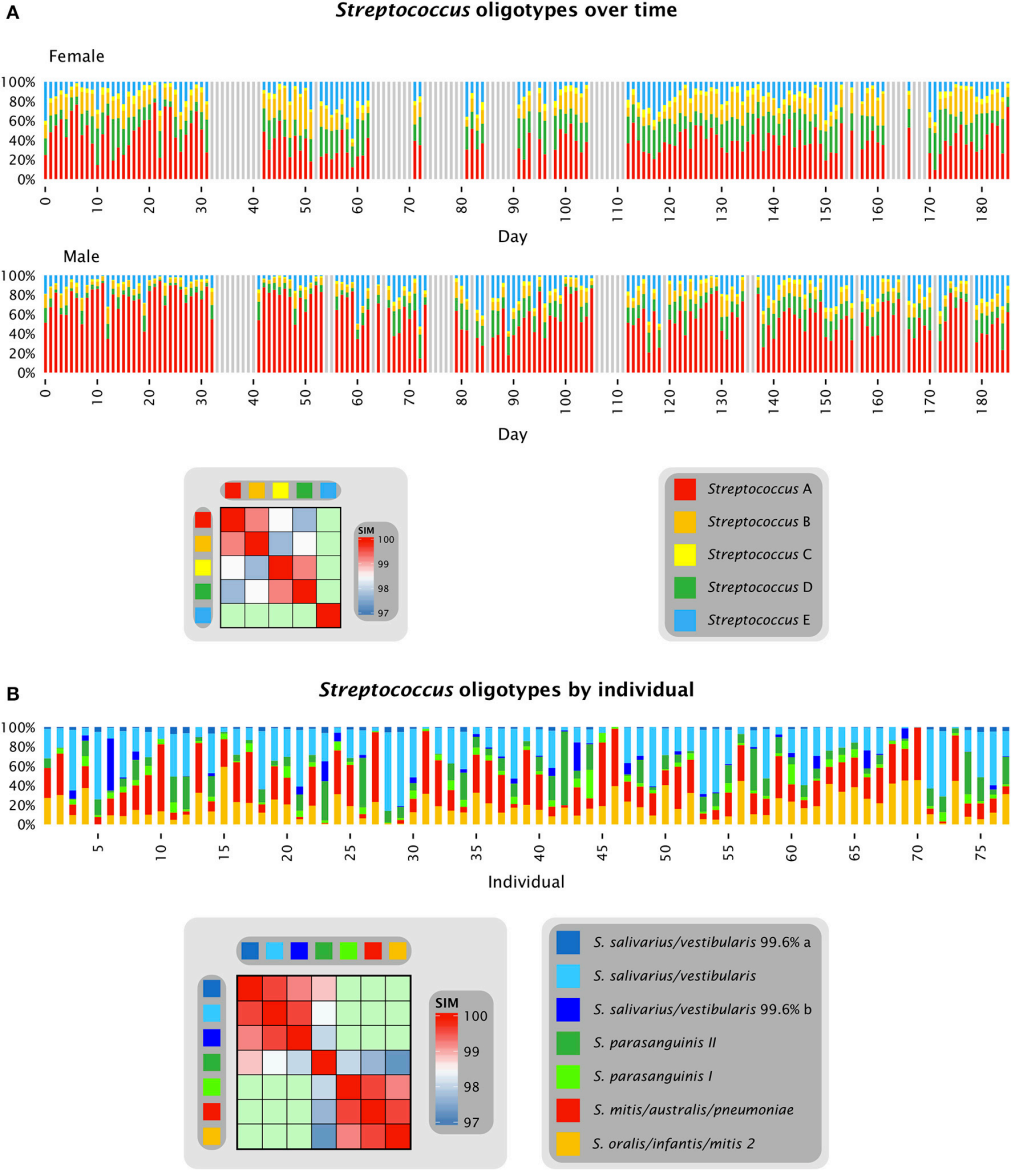
***Streptococcus* oligotypes in the tongue**. **(A)** Relative abundance of *Streptococcus* oligotypes in time series data from the tongue. Oligotypes with at least 0.5% mean abundance in at least one of the two individuals are shown. Colored bars represent the relative abundance of each of the five oligotypes in a single sample; data shown is for days 0 to 185 and gray bars represent days for which no data is available. The heat map shows the similarity of each pair of oligotypes. *Streptococcus* A (oligo_003 in Table S1) is identical to the reference sequences for 6 species in HOMD including *S. mitis, S. mitis* biovar 2, *S. infantis, S. oralis*, and *Streptococcus spp*. HOT 070 and 071; *Streptococcus* B (oligo_008) is identical to the reference sequences for 7 species in HOMD including *S. parasanguinis I, S. parasanguinis II, S. australis*, and *Streptococcus spp*. HOT 057, 065, 066, and 067; *Streptococcus* C (oligo_012) is identical to the reference sequences for *S. peroris* and *Streptococcus spp*. HOT 068 and 074; *Streptococcus* D (oligo_027) is identical to the reference sequences for *S. cristatus, S. gordonii, S. sinensis, S. oligofermentans*, and *Streptococcus spp*. HOT 056 and 069; and *Streptococcus* E (oligo_006) is identical to the reference sequences for *S. salivarius* and *S. vestibularis*. *Streptococcus* A, B, C, and D are all within 97% identity of one another as shown by the heat map. **(B)** Relative abundance of *Streptococcus* oligotypes in HMP data from the tongue, sequenced in the V1-V3 region. Oligotypes with at least 0.5% mean abundance across all sampled individuals are shown, and are assigned the name of the closest match in HOMD; where the closest match is not 100% identical, the percent identity is shown. In addition to the taxa listed in the key, the oligotype identified as *S. oralis/infantis/mitis biovar 2* is also identical to *Streptococcus spp*. HOT 055, 058, 061, and 070 and the oligotype identified as *S. mitis/australis/pneumoniae* is also identical to *Streptococcus spp*. HOT 070, 071, and 074. Data for **(B)** are from Eren et al. (2014).

**Figure 4:**
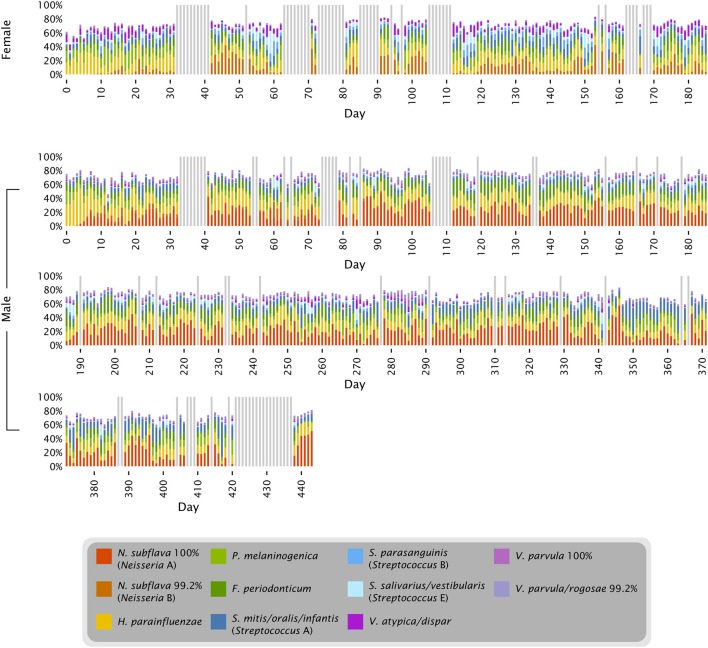
**Time series of abundant oligotypes**. The 11 oligotypes shown include the 8 most abundant in each subject; 5 oligotypes are in the top 8 in both subjects. Colored bars represent the abundance of each oligotype in each sample; gray bars represent days for which no data is available.

The stable dominance of one oligotype of *Neisseria* in each individual, relative to the other *Neisseria* oligotypes, occurred in a context of rapid fluctuation in the abundance of *Neisseria* and all other taxa as a proportion of the total community. The overall behavior of the system was a dynamic equilibrium with rapid fluctuations in relative abundance but without long-term directionality, as shown in Figures 3, 4. Figure 3 shows the relative proportions of the five most abundant *Streptococcus* oligotypes over time in each individual. The most abundant *Streptococcus* oligotype overall, labeled *Streptococcus* A in the figure, is identical to *S. mitis, S. oralis*, and *S. infantis* in the V4 region; this oligotype ranged in abundance from 9 to 75% of the *Streptococcus* in the female subject and from 10 to 92% of the *Streptococcus* in the male (Figure 3A). Relative abundance of taxa not only ranged widely but also changed quickly as seen, for example, in samples 269 and 270 from the male subject, in which the relative abundance of *S. mitis/oralis/infantis* dropped from 78 to 10% of the *Streptococcus* in the sample over the course of a single day (Table S1). For comparison, the corresponding oligotypes identical to *S. mitis, S. oralis*, and *S. infantis* sampled from the tongue dorsum of multiple individuals from the HMP together ranged from 1 to approximately 90% of the *Streptococcus* genus on the tongue (Figure 3B and Eren et al., 2014). Thus, a substantial fraction of the range of variability observed across individuals was also observed within a single individual over time.

The proportions of *Neisseria* and *Streptococcus* can be seen in the context of other major tongue dorsum oligotypes in Figure 4. The major oligotypes shown in the figure each ranged from double-digit abundance to near-absence in samples over the course of the time series. The wide fluctuations in sample composition within an individual raised the question of the significance of differences between individuals compared to the variation that exists within an individual over time. OTU-level analysis of the tongue dorsum time course data showed that between-subject UniFrac distances were greater than within-subject distances (Caporaso et al., 2011), and likewise OTU-level analysis of HMP data showed between-subject differences within a body site greater than within-subject differences (Human Microbiome Project Consortium, 2012). Such inter-individual differences are also reflected in our oligotyping analysis, in the form of sometimes widely differential mean abundances of oligotypes between the two individuals, such as a greater abundance of *Neisseria* in the male and a greater abundance of several *Streptococcus* oligotypes in the female (Figures 3, 4, and Table S1). These differences are concrete examples of the underlying taxon composition that leads to higher community dissimilarity scores between than within individuals. However, we wondered whether the summary statistic of average community dissimilarity was obscuring the magnitude of the shifts in community composition within individuals over time and giving a misleading impression about the relative importance of differences between and within individuals.

**Figure 5:**
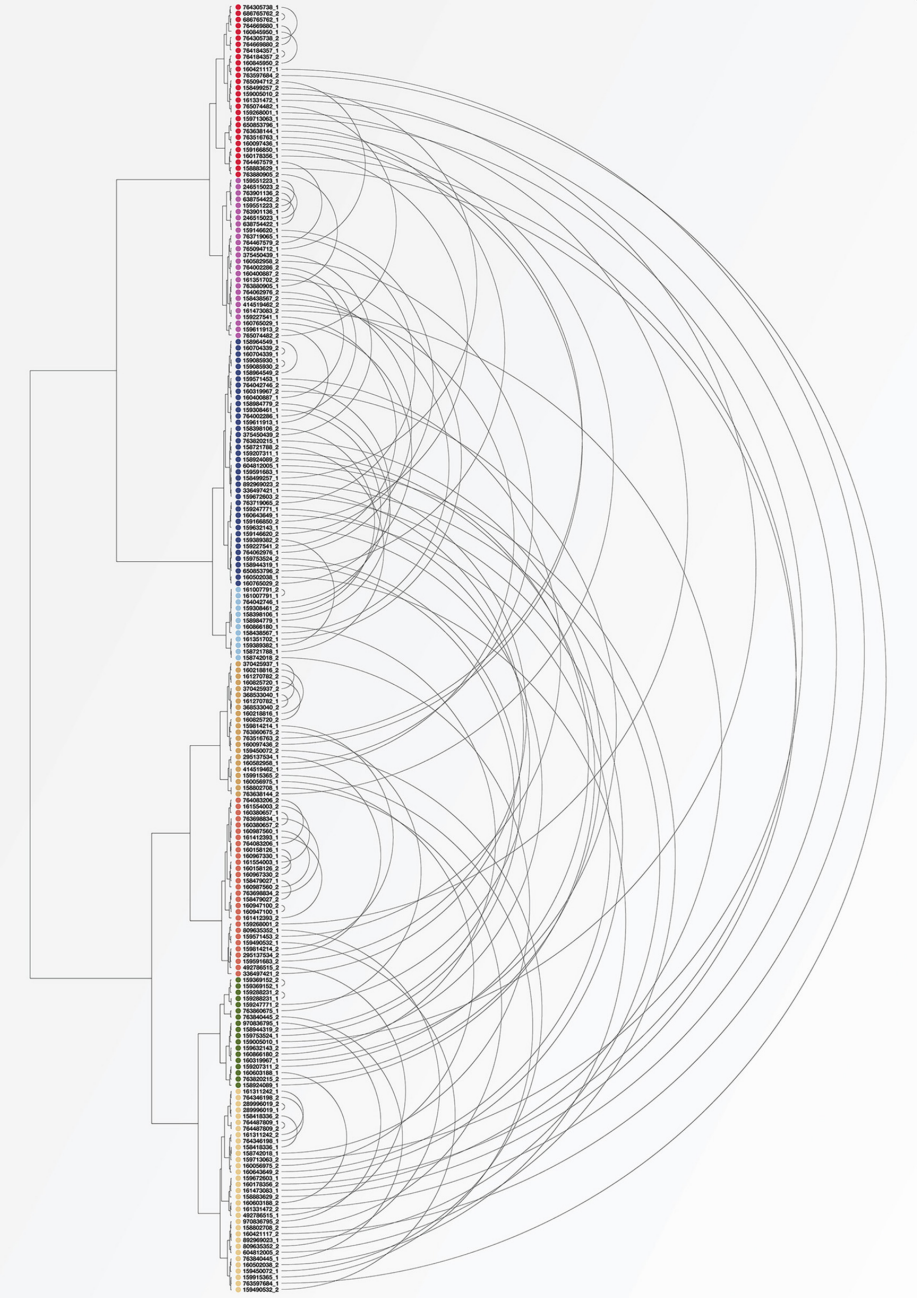
**Cluster analysis of individuals sampled by HMP at two visits**. Each dot represents a tongue dorsum sample from one of 104 individuals sampled at two visits at least 30 days apart. Samples were classified to genus and clustered using the Morisita-Horn dissimilarity index. Arcs connect the two samples from each subject. Short arcs indicate subjects whose community composition was similar at the two visits; long arcs indicate subjects whose second sample was substantially different in composition from the first.

For a quantitative comparison of variation within and between subjects in these different studies, standard beta-diversity indices are not calculable because the studies analyzed different regions of the 16S rRNA gene and employed different amplification and sequencing protocols. However, some of the HMP subjects were sampled on more than one visit, affording the opportunity to assess the variability over time in these individuals compared to variation between subjects measured using the same study protocol. To make this comparison we identified 104 subjects for whom tongue dorsum samples from the V3-V5 region were available from two different visits, which were separated by 30–359 days (Aagaard et al., 2013). Reads in these samples had previously been trimmed and classified to genus using standard HMP pipelines (The Human Microbiome Project Consortium, 2012). Using data on the number of reads classified into each genus for each of these samples, we carried out a cluster analysis using the Morisita-Horn dissimilarity index. Figure 5 shows the resulting clusters. For each of the 104 subjects, the two samples from different time points are connected by an arc. As can be seen in the figure, for some subjects the two samples from different time points cluster tightly together (short arcs), but for many subjects the two samples are located in different clusters (long arcs). These clusters can be related back to the taxon composition of each sample; for example, the cluster colored in light blue consists of samples that are more than 50% *Streptococcus* while samples in the cluster shown in red have a high proportion of *Fusobacterium*. This analysis supports the conclusion that many of the apparent microbiome differences between individuals seen in the HMP data are a result not of stable differences from person to person, but of the limited information that results from “snapshot” sampling a continuously changing system (an individual tongue) at a single time point.

### Correlated abundance between members of different genera

The time series abundance data permit an assessment of the degree of correlation or anti-correlation in the abundance of individual oligotypes. Such an assessment would provide a basis for inferring significant biological associations of taxa. Remarkably, the data showed strong correlations between pairs of oligotypes both within a taxon and across taxa (Figure S1).

**Figure 6:**
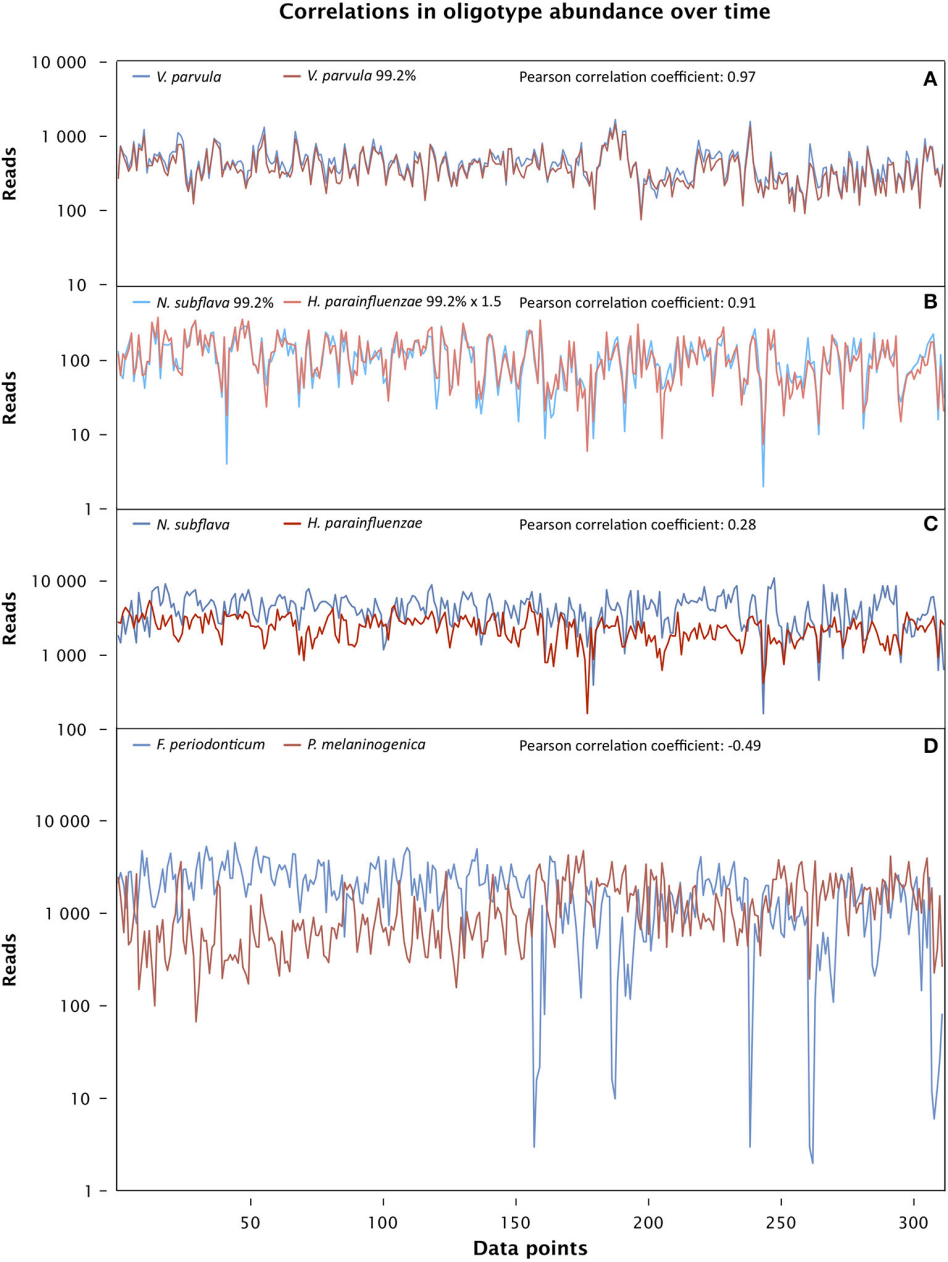
**Time series correlation analysis**. The abundance of each oligotype, measured in reads, is plotted for each sample from day 66 through 420 in the male subject, and the Pearson correlation coefficient for each pair of oligotypes over this data range is shown above the plot. **(A)** Two strongly correlated oligotypes matching the taxon *Veillonella parvula* (oligo_007 and oligo_009 in Table S1) (*p* << 0.0001). **(B)** Strongly correlated oligotypes 99.2% identical to *Neisseria subflava* (oligo_024) and *Haemophilus parainfluenzae* (oligo_030) (*p* << 0.0001). **(C)** Two oligotypes identical to *Neisseria subflava* (oligo_001) and *Haemophilus parainfluenzae* (oligo_002) are weakly correlated (Pearson, *p* = 0.18 after Bonferroni correction; Spearman, *p* < 0.01 after Bonferroni correction). **(D)** Oligotypes identical to *Fusobacterium periodonticum* (oligo_004) and *Prevotella melaninongenica* (oligo_005), showing moderate anti-correlation (*p* << 0.0001).

The strongest correlation was between two oligotypes that are among the 10 most abundant in the dataset and whose best match in HOMD is to the same taxon, *Veillonella parvula*. One, oligo_007, is identical to the *V. parvula* reference sequence and the other, oligo_009, differs from it by a single nucleotide (Figure 6A). The strength of their correlation suggests either that they are in an extraordinarily close symbiosis or that they represent two distinct rRNA genes present in the same cell. One advantage of the oral microbiome as a study subject is the presence of sequenced genomes for a high fraction of oral microbial taxa (Dewhirst et al., 2010), allowing a direct test of the possibility that any two given rRNA genes are present in a single organism. We carried out a BLAST search of genomic DNA using the HOMD web site (HOMD.org) and found both *V. parvula* oligotypes in the sequenced genome of *V. parvula* DSM 2008/ATCC 10790. We conclude that the two tightly correlated *V. parvula* oligotypes represent two sequences found in the same organism.

In contrast to the *V. parvula* oligotypes, another strongly-correlated pair of oligotypes (oligo_024 and oligo_030) represent species in different taxonomic families: one member of the pair differs by a single nucleotide from the *Haemophilus parainfluenzae* reference and the other differs by a single nucleotide from the *Neisseria subflava* reference (Figure 6B). Partially or completely sequenced genomes are available for *N. subflava* as well as the related taxa *N. flavescens* and *N. mucosa*, and for *H. parainfluenzae* as well as the related *H. influenzae* and *H. haemolyticus*, among others. BLAST searches revealed that the *H. parainfluenzae* oligotype oligo_030 is no more than 87% identical to any region of any sequenced *Neisseria* genome, while the reverse is true for the *N. subflava* oligotype oligo_024: it is no more than 87% identical to any sequenced *Haemophilus* genome. We conclude that these two oligotypes reside in different organisms, and their strong correlation reflects either a close symbiotic interaction between them, or strong specialization of both organisms to the same micro-habitat.

The abundance traces of the two pairs of oligotypes shown in Figures 6A,B are nearly identical to those obtained by other investigators who analyzed the same dataset using an entirely different method aimed at identifying biologically meaningful units with single-nucleotide resolution (Tikhonov et al., 2014). This similarity supports the general validity of both methods. However, we reach opposite conclusions concerning which of these pairs is made up of sequences present in the same genome and which are in different genomes. We conclude based on whole-genome sequences that the two *Veillonella* sequences are in the same genome and that the *Haemophilus* and *Neisseria* sequences are in different cells. Tikhonov et al. confined their analysis to the sequences *per se*. Based on autocorrelation coefficients, they concluded that the two sequences which we identify as *Veillonella* are at least partially contributed by different cells and that the sequences we identify as *Haemophilus* and *Neisseria* likely originate from the same cells. We believe our conclusions benefit from the genome-mining and cross-referencing to HOMD, but future work is necessary to determine which conclusion is correct.

The most abundant oligotypes in the tongue time series dataset are not strongly correlated with one another. For example, the two most abundant oligotypes in the dataset, which are identical to the HOMD reference sequences for *N. subflava* and *H. parainfluenzae*, each make up more than 10% of the entire dataset and have abundance distributions that are weakly correlated with each other (Figure 6C and Figure S1). The weak correlation of these highly abundant oligotypes contrasts with the tight correlation of their lower-abundance variants discussed above and suggests differences in the underlying biology of the high- and low-abundance types. Possibly, the high-abundance oligotypes represent generalist organisms that do not require specialized habitat or tight taxon-taxon associations. Alternatively, the more abundant oligotypes may encompass a heterogeneous collection of organisms with identical V4 regions of the 16S rRNA gene but with distinctive habitat requirements.

Additional, moderately positive correlations exist among pairs of oligotypes from widely different taxa such as *Streptococcus, Haemophilus*, and *Alloprevotella* (Figure S1) and likely result from a preference for similar habitats or environmental conditions. In contrast, the fourth and fifth most abundant oligotypes overall, whose sequences are identical to the *Fusobacterium periodonticum* and *Prevotella melaninogenica* reference sequences, are moderately anticorrelated (Figure 6D); this anticorrelation could result from an active antagonism between two taxa or from a preference for incompatible microhabitats. In sum, correlation analysis of the time series data provides strong indications of possible functional or habitat associations among diverse taxa.

### Are the fluctuations in oligotype abundance periodic?

Casual inspection of the time series data gives the impression that the oligotype fluctuations could be periodic. One possible hypothesis for periodic variation in the composition of the tongue microbiome is a periodic variation in host activity such as might occur over weekends as opposed to the workweek. We tested for reproducible periodicity in the data by carrying out auto-correlation and Fourier transform analysis for each oligotype. Auto-correlations were evaluated over a window of plus or minus 21 days. Consistent with the observation of rapid fluctuations, the auto-correlation signal was strongest for a one-day time lag, which agrees with the results of Tikhonov et al. (2014). However, no consistent signal was observed for any of the abundant oligotypes either with auto-correlation or with Fourier analysis that would suggest a weekly or other periodicity. A few minor oligotypes showed a weak signal corresponding to weekly periodicity but the signal was not of sufficient magnitude to admit of a strong conclusion. Proper evaluation of such a possibility will require a directed investigation.

### A characteristic tongue microbiota

**Figure 7:**
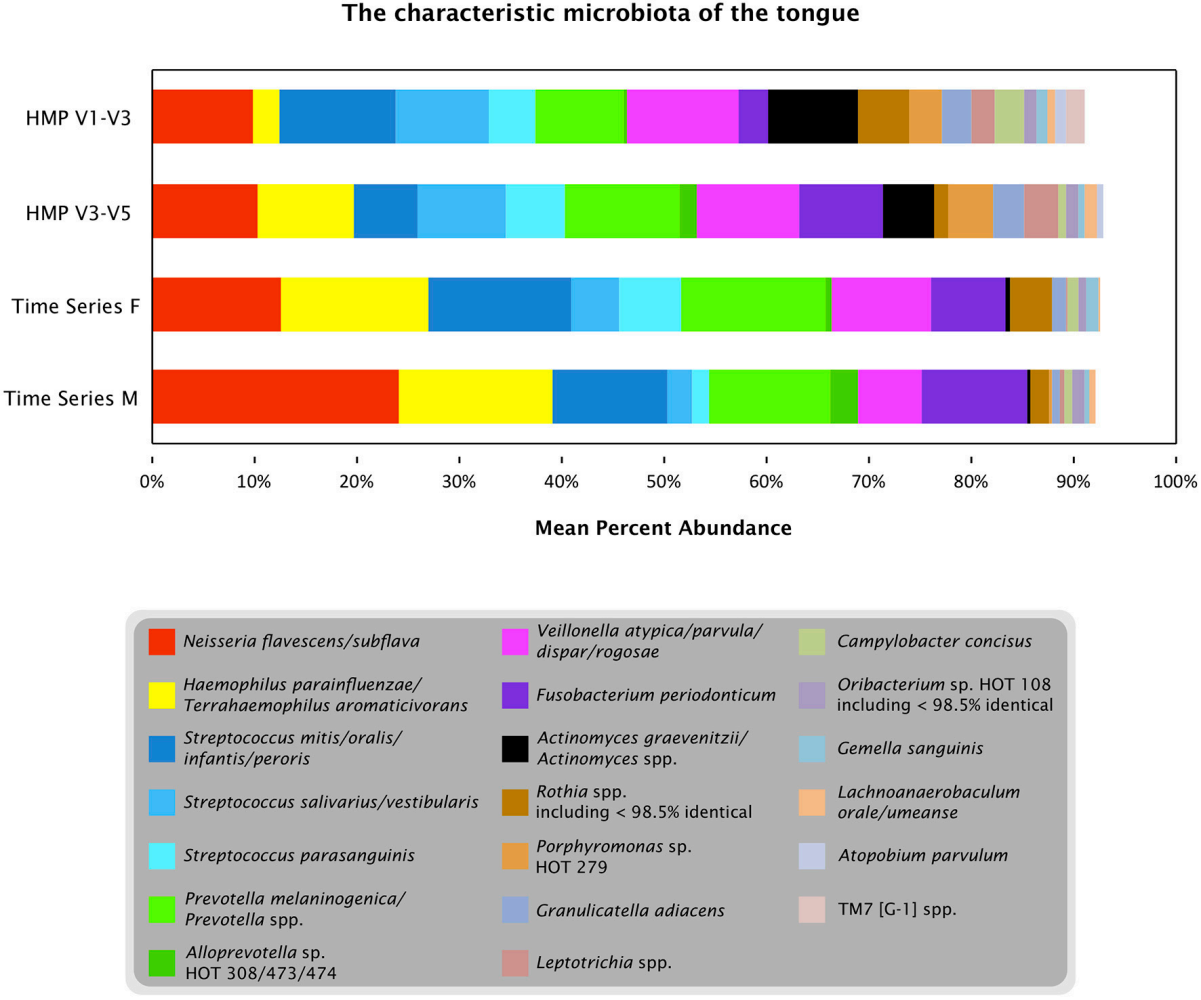
**The characteristic microbiota of the tongue**. Colored bars represent the mean abundance of the taxa that consistently occur in the time series and HMP datasets. For each of the taxa shown, the HOMD reference sequence for the taxon is at least 98.5% identical to one or more oligotypes that make up at least 0.5% of the time series tongue data (Female, Male) and/or the V3-V5 and V1-V3 tongue dorsum data from the HMP. Exceptions to the 98.5% identity criterion (*Rothia* spp. and *Oribacterium* sp. HOT 108) indicate cases where a precisely matched reference sequence may not yet be represented in HOMD.

Oligotyping three datasets from the tongue (one time course and two broad samplings of individuals) showed that a limited number of species-level or near-species-level taxa consistently make up the majority of the microbiota on the tongue. Detailed taxonomic comparison of oligotypes across these datasets is not straightforward, because different regions of the 16S rRNA gene were sequenced in each case: V4 for the time course data and V1-V3 and V3-V5 for the HMP data. Nonetheless, taxonomic assessments can be made by comparing each sequence to a curated reference database, the HOMD, and using the matching reference sequence(s) as an estimate of the taxonomy of the oligotype. Twenty such reference sequences, or groups of closely related reference sequences, collectively account for 91–93% of the reads from each dataset (Figure 7). Eighteen of these 20 were detected in every sample or nearly every sample from both individuals in the time series (Table S1). Thus, while the temporal core microbiome in this dataset is composed of only a small fraction of the taxa that are detectable (Caporaso et al., 2011), this temporal core nonetheless constitutes the majority of the organisms on the tongue.

In contrast to these similarities, there are also differences among the abundant taxa present in the time series compared to the HMP data. Several taxa are relatively depauperate in the time series data set compared to HMP, including *Actinomyces spp*., *Leptotrichia spp*., and *Porphyromonas sp*. HOT 279; these differences may reflect true characteristics of the microbiomes of the sampled individuals, or may result from primer bias or other technical differences in experimental procedures. The genus *Rothia* is represented in all three datasets but the oligotypes representing this genus do not match the same species consistently across datasets. This inconsistency may be explained by technical or biological differences, but an alternative possibility is that sequences from this genus represent one or a few taxa that are consistently present across datasets but have 16S rRNA gene sequences divergent from the reference sequences currently represented in HOMD. For such a taxon the closest match in the V1-V3 region may be to one reference sequence; in the V4 region its closest match may be to a different reference sequence, and these differences in taxonomic assignment in the different regions may obscure the consistency with which the identical taxon is present across datasets.

## Discussion

### Microbial community dynamics followed using oligotyping

Understanding the forces that shape microbial communities in the human microbiome requires following dynamic changes in these communities over time. Rapid decreases in the cost of DNA sequencing have made it possible to generate the large amounts of data required for studies of dynamics, but analysis methods limited to the genus or OTU level have limited the opportunities for analyzing the dynamics within a single species or between closely related species. This study provides an example of the single-nucleotide taxonomic resolution of oligotyping which, in turn, enables analysis of microbial dynamics and associations that would otherwise not be possible if taxa were lumped into heterogeneous groups.

### Phase transitions of oligotypes

Our observation of changing relative abundance of *Neisseria* oligotypes on the tongues of two different individuals showed that in these instances, replacement of an initially dominant oligotype occurred over a time scale of days, and the newly dominant type remained dominant for the rest of the months-long sampling period. Thus the period of transition was relatively abrupt in comparison to the duration of the subsequent dominant phase. The causes both of the replacement, and of the stable dominance, remain uncertain. After the first few days of sampling the two oligotypes that became dominant were different in the two individuals but were detected in nearly every sample from each of them. It is possible that these two oligotypes newly invaded the tongue habitat of these individuals near the beginning of the time course and, once present, proliferated in what was for them a favorable environment. Alternatively, it is possible that they were present but simply below the detection limit for the first few days, and their sudden proliferation was caused by changes in the oral environment or the surrounding microbiota, changes perhaps occasioned by the daily sampling itself. In both individuals the oligotype that was not dominant nevertheless persisted in low abundance, showing that (unsurprisingly for the oral environment) dispersal is not the limiting factor regulating the abundance of these taxa in a given mouth. The dynamics displayed by these oligotypes are similar to the behavior of some closely-related 97% OTUs in a time series of gut and saliva samples from two individuals (David et al., 2014), in which rapid transitions are followed by extended periods of stable dominance of one of the OTUs. A similar pattern was also observed in the within-species dynamics of *Staphylococcus epidermidis* in a time series from the gut microbiome of a premature infant (Sharon et al., 2013) in which the changes in strain abundance were at least partially attributable to the dynamics of infecting bacteriophage. The extended dominance periods we observe are difficult to explain as a consequence of phage-driven dynamics, however, unless one invokes development of host resistance or changes in phage infectivity (Sharon et al., 2013) or the presence of multiple strains that have different virus sensitivities and that are succeeding one another, but which are indistinguishable in 16S rRNA gene sequence (Fuhrman, 2009) and thus undetectable with this data.

### Implications of high variability in taxon relative abundance over time

The high variability and rapid change in microbial communities in the time series data set were noted by Caporaso et al. (2011) as well as the contribution of blooms of particular genus-level taxa to the dissimilarity of the overall community over time. Our oligotyping results extend these findings to the species- or near-species level, as shown in the example of *Streptococcus* in which dramatic changes occur in the relative, as well as the absolute, abundance of each oligotype as a proportion of the genus abundance over time. From our analysis of the HMP data for the *Streptococcus* community of many individuals at a single time point, it was evident that a number of major *Streptococcus* taxa were present in every individual; however it was not possible to determine whether their abundances fluctuated over time or whether communities in some individuals were strongly and continually biased in favor of one or another taxon. Our results with the time-series data for the tongue dorsum suggest that a substantial portion of the variation in taxon abundance occurring between individuals in the HMP data can be explained by the temporal variation of abundance within individuals.

This high variability has implications for the fine-scale spatial and metabolic structure of the tongue flora. Given our observation of a consistent, characteristic tongue dorsum microbiota over time and across individuals, one could hypothesize that these taxa comprise a tightly integrated community with finely tuned metabolic interactions with one another and with cells of different microbial species intimately intermingled at micron scales in a relatively constant stoichiometry. The high overall variability in relative abundance among these taxa, however, argues against such a hypothesis. Rather, the microbiota likely constitute a number of distinct assemblages occupying different spatial positions, preferring different environments, or succeeding one another over time. Certain subsets of the assemblage that show correlated distribution, such as the oligotypes identified with *H. parainfluenzae* and members of the *N. subflava* group, may constitute a functional unit. Other anti-correlated subsets, however, such as the oligotypes identified with *F. periodonticum* and *P. melaninogenica*, may reflect that the corresponding taxa interact in an antagonistic fashion or that they prefer different environmental conditions.

The reasons underlying the large fluctuations in relative abundance across taxa are an interesting question for further study. Disturbances caused by oral hygiene procedures and ingestion of food or liquids occur with higher frequency than the observed community fluctuations and are unlikely to be the sole driver of these fluctuations. For an assemblage residing on a shedding epithelial surface, the sporadic availability of new surfaces for colonization may give a temporary advantage to taxa that are more effective initial colonizers or may be, by chance, spatially well-positioned to colonize new habitat. Alternate explanations include changes in activity of the host immune system, diurnal physiological changes, the dynamics of bacteriophage populations, competition, or stochastic variation. Sporadic changes in host behavior may also be responsible.

### The use of HOMD to connect oral oligotype datasets

Short regions of the rRNA gene have limitations for high-resolution identification and differentiation of microbes. Potential confusion arises when taxa of interest are differentiated by only one or a few nucleotides in the sequenced region, but these limitations can be mitigated by making use of taxonomic information to relate distinct datasets to one another.

An example in the data shown here is the time series oligotype labeled *Streptococcus* D (Figure 3A). This oligotype is identical in the V4 region to the HOMD reference sequence for *S. gordonii* but is also only a single nucleotide different from the HOMD reference sequence for *Streptococcus parasanguinis*. Additional information about the likely taxonomy of this oligotype comes from the HMP datasets from V1-V3 and V3-V5; neither of these datasets shows a significant contribution of *S. gordonii* to the tongue microbiota, while both show a substantial contribution of *S. parasanguinis* (Figure 3B). Evaluation of the time series data in the context of the HMP data therefore suggests that *Streptococcus* D is more accurately identified as a variant of *S. parasanguinis*. Similar considerations apply to the *Neisseria* oligotypes (Figure 1). The species *N. flavescens, N. subflava*, and *N. flava* form a phylogenetically distinct group according to whole-genome sequence data (Bennett et al., 2013) and are shown by HMP data to be important in the tongue microbiota (Eren et al., 2014). In the V4 region there are only 1 or 2 nucleotide differences between these taxa, leading to ambiguity in identification of the time series oligotypes in the absence of additional information. This information can be found in HMP data: using V1-V3 sequences, the abundant oligotypes of this group are unequivocally identified as most similar to *N. flavescens*, which differs from *N. flava* and *N. subflava* by 11 nucleotides in the oligotyped region of V3. These two examples demonstrate the power of a well-curated database and applying multiple lines of evidence to the identification of taxa.

### The core tongue microbiome

With the species-level description of its consistent core microbiome that we present here, the tongue becomes one of only a small number of habitats for which a numerically abundant core microbiome has been described at the species level. Our results support the conclusions of Kraal et al. (2014) who analyzed whole-genome shotgun samples from the HMP and concluded that the species *Veillonella dispar* was abundant in every tongue microbiome sampled and that three other species (*S. parasanguinis, S. salivarius*, and *P. melaninogenica*) each were abundant in at least 87% of tongues sampled. Given the close similarity of the microbiomes of the tongue and of saliva (Mager et al., 2003; Eren et al., 2014) it is not surprising that the set of genus-level taxa detected in all or nearly all saliva samples by Stahringer et al. (2012) is also concordant with our set of core taxa, as is the set of genera found in all saliva samples by Lazarevic et al. (2010). The presence of a consistent core tongue microbiota argues against the idea that many functions in the overall oral microbial community can be carried out by any one of a number of interchangeable taxa, and argues instead for the presence of niche specialists whose role is not readily filled by alternative taxa (Fuhrman, 2009). The relative simplicity of the core tongue microbiota contrasts with the hundreds of taxa that are described from the mouth as a whole (Dewhirst et al., 2010), many of which are specialized to a subset of habitats within the mouth (Eren et al., 2014). It may be a general characteristic of microbial ecosystems to appear enormously complicated when considered at spatial scales that lump together disparate habitats, but to resolve into more tractable communities when the habitat is accurately and narrowly defined.

### Making full use of the information in high-throughput sequencing data sets

There is a growing recognition that high-throughput sequencing data contains information that is not fully expressed by partitioning the data into conventional OTUs. Some form of partitioning is necessary because both neutral variation in natural populations and sequencing errors create a profusion of sequence variants without underlying biological meaning. However, OTUs that are defined purely by a threshold of sequence similarity are phylogenetically and ecologically heterogeneous and inconsistent (Prosser et al., 2007; Schloss and Westcott, 2011; Koeppel and Wu, 2013; Schmidt et al., 2014). Alternative approaches make use of the fact that the noise arising from neutral variation and sequencing errors is randomly distributed with respect to ecology. For example, an approach termed “distribution-based clustering” employs information about the distribution of sequences among habitats or samples to differentiate noise from meaningful variation and thus inform the definition of taxonomic units (Preheim et al., 2013). In a “denoising” approach (Tikhonov et al., 2014), sequencing error and temporal cross-correlation were analytically distinct but temporal cross-correlation analysis was used to determine which unique sequences were “real,” i.e., not attributable to noise.

Oligotyping is an information theory-based approach that employs Shannon entropy to identify nucleotide positions of high variation within a dataset (Eren et al., 2013), thereby distinguishing meaningful variation from sequencing errors (Huse et al., 2007; Minoche et al., 2011). Like the cross-correlation approaches, the Shannon entropy method has the capacity to discriminate among closely related taxa at the sub-species level. However, unlike these other approaches, the Shannon entropy method partitions the data into oligotypes independent of cross-sample correlations. This independence means that habitat or temporal correlation analysis can be employed at a later stage in data analysis, providing an independent way of assessing the biological meaning and distinctiveness of sequence variants.

For the human oral microbiome, the presence of a highly curated database and a large number of sequenced genomes provides an additional layer of analytic power. Sequence differences that rise above the level of noise, as identified by oligotyping or cross-correlation, can be associated with known taxa via the HOMD, allowing the comparison of data across datasets even when different regions of the 16S rRNA gene were employed for sequencing. Distinguishing whether oligotypes represent different 16S rRNA genes within a single organism or are tags for different organisms is enabled by access to full genomes. This cross-dataset analysis and genome-mining capability greatly expands the usefulness of datasets. In summary, we have used high-resolution taxonomic analysis of high-throughput time series data to provide insight into the microbial population dynamics of the tongue. Our results have revealed phase transitions of closely related taxa and unanticipated associations of taxa from different genera. We expect that our approach will permit future, targeted analyses of specific microbial interactions and dynamics.

## Author contributions

Jessica L. Mark Welch, A. Murat Eren, and Gary G. Borisy conceived and designed the work; Jessica L. Mark Welch, Daniel R. Utter, Blair J. Rossetti, David B. Mark Welch, and A. Murat Eren analyzed data; Jessica L. Mark Welch and Gary G. Borisy wrote the paper; and all authors reviewed, edited, and approved the final manuscript.

### Conflict of interest statement

The authors declare that the research was conducted in the absence of any commercial or financial relationships that could be construed as a potential conflict of interest.
