# Supplementary material for: Dynamics of tongue microbial communities with single-nucleotide resolution using oligotyping
Source: Front Microbiol. 2014 Nov 7;5:568. doi: 10.3389/fmicb.2014.00568 (PMC4224128; doi:10.3389/fmicb.2014.00568)
Supplement: Supplementary file 2 [file Image1.PDF]

Supplementary Figure S1

	S. mitis/oralis/infantis	H. haemolyticus/influenzae	A. sp. (HOT-473)	P. nigrescens 98.5%	A. sp. (HOT-474)	P. sp. (HOT-299) 98.5%	P. sp. (HOT-299) 99.2%	P. salivae	P. melaninogenica	V. atypica/dispar	P. sp. (HOT-313)	N. subflava 99.2% (oligo_011)	N. pharyngis/mucosa/flava	H. parainfluenzae	N. subflava	N. subflava 99.2% (oligo_024)	H. parainfluenzae 99.2%	F. periodonticum	C. concisus 99.2%	L. umeaense	O. sp. 96.9%	V. parvula	V. parvula 99.2%	S. parasanguinis	S. salivarius/vestibularis	R. aeria 95.4%	S. cristatus/gordonii/sinensis	G. sanguinis	G. adjacens	S. peroris
S. mitis/oralis/infantis	1.00	0.62	0.56	0.00	0.19	0.26	0.13	0.14	0.30	-0.03	-0.08	-0.24	0.20	-0.41	-0.37	-0.33	-0.40	-0.47	-0.19	-0.37	-0.32	-0.30	-0.30	-0.22	-0.17	-0.26	0.18	0.00	-0.06	0.05
H. haemolyticus/influenzae	0.62	1.00	0.62	0.16	0.34	0.44	0.37	0.30	0.32	-0.13	0.05	-0.12	0.29	-0.37	-0.29	-0.15	-0.33	-0.29	-0.06	-0.27	-0.32	-0.42	-0.42	-0.38	-0.28	-0.41	-0.12	-0.23	-0.39	-0.28
A. sp. (HOT-473)	0.56	0.62	1.00	0.16	0.43	0.52	0.33	0.12	0.28	-0.15	-0.08	-0.14	0.22	-0.31	-0.32	-0.18	-0.29	-0.31	-0.14	-0.31	-0.31	-0.34	-0.36	-0.35	-0.25	-0.39	-0.10	-0.21	-0.34	-0.27
P. nigrescens 98.5%	0.00	0.16	0.16	1.00	0.52	0.63	0.57	0.45	0.42	0.06	0.29	-0.03	-0.01	-0.29	-0.28	-0.23	-0.31	-0.03	0.06	-0.07	-0.15	-0.04	-0.04	-0.28	-0.13	-0.43	-0.26	-0.22	-0.26	-0.32
A. sp. (HOT-474)	0.19	0.34	0.43	0.52	1.00	0.67	0.60	0.10	0.22	-0.25	-0.03	-0.06	-0.01	-0.09	-0.16	0.07	-0.09	-0.11	-0.20	-0.22	-0.39	-0.29	-0.33	-0.52	-0.42	-0.47	-0.39	-0.37	-0.33	-0.39
P. sp. (HOT-299) 98.5%	0.26	0.44	0.52	0.63	0.67	1.00	0.83	0.26	0.52	-0.21	0.00	-0.01	0.04	-0.28	-0.20	-0.05	-0.23	-0.24	-0.19	-0.31	-0.40	-0.23	-0.28	-0.47	-0.37	-0.52	-0.25	-0.35	-0.34	-0.34
P. sp. (HOT-299) 99.2%	0.13	0.37	0.33	0.57	0.60	0.83	1.00	0.19	0.50	-0.21	-0.03	0.09	-0.02	-0.21	-0.09	0.02	-0.14	-0.20	-0.17	-0.23	-0.37	-0.22	-0.25	-0.46	-0.33	-0.49	-0.32	-0.36	-0.33	-0.35
P. salivae	0.14	0.30	0.12	0.45	0.10	0.26	0.19	1.00	0.66	0.56	0.57	-0.16	0.07	-0.44	-0.51	-0.49	-0.52	-0.26	0.21	-0.02	-0.12	0.03	0.10	-0.03	0.07	-0.30	0.02	-0.14	-0.15	-0.24
P. melaninogenica	0.30	0.32	0.28	0.42	0.22	0.52	0.50	0.66	1.00	0.38	0.20	-0.11	0.09	-0.46	-0.46	-0.41	-0.48	-0.50	-0.10	-0.25	-0.34	0.04	0.07	-0.20	-0.07	-0.38	0.08	-0.08	-0.17	-0.19
V. atypica/dispar	-0.03	-0.13	-0.15	0.06	-0.25	-0.21	-0.21	0.56	0.38	1.00	0.51	-0.03	0.06	-0.36	-0.39	-0.48	-0.41	-0.25	0.22	0.09	0.12	0.36	0.47	0.35	0.45	0.10	0.25	0.21	0.14	0.03
P. sp. (HOT-313)	-0.08	0.05	-0.08	0.29	-0.03	0.00	-0.03	0.57	0.20	0.51	1.00	-0.03	0.11	-0.23	-0.37	-0.37	-0.36	-0.03	0.43	0.19	0.07	0.10	0.14	0.09	0.19	-0.11	0.00	-0.11	-0.05	-0.17
N. subflava 99.2% (oligo_011)	-0.24	-0.12	-0.14	-0.03	-0.06	-0.01	0.09	-0.16	-0.11	-0.03	-0.03	1.00	0.11	0.01	0.27	0.11	0.14	0.11	0.07	0.05	0.12	0.06	0.06	0.08	0.02	0.10	-0.03	0.00	-0.10	-0.16
N. pharyngis/mucosa/flava	0.20	0.29	0.22	-0.01	-0.01	0.04	-0.02	0.07	0.09	0.06	0.11	0.11	1.00	-0.08	-0.24	-0.17	-0.15	-0.28	0.02	-0.15	-0.06	-0.21	-0.18	-0.07	-0.01	-0.06	0.09	-0.05	-0.07	-0.11
H. parainfluenzae	-0.41	-0.37	-0.31	-0.29	-0.09	-0.28	-0.21	-0.44	-0.46	-0.36	-0.23	0.01	-0.08	1.00	0.26	0.70	0.79	0.01	-0.28	-0.15	-0.18	-0.15	-0.20	-0.25	-0.30	0.25	-0.30	-0.20	0.04	-0.05
N. subflava	-0.37	-0.29	-0.32	-0.28	-0.16	-0.20	-0.09	-0.51	-0.46	-0.39	-0.37	0.27	-0.-24	0.26	1.00	0.60	0.58	0.36	-0.16	0.11	0.07	-0.09	-0.12	0.02	-0.16	0.26	-0.13	0.02	0.17	0.14
N. subflava 99.2% (oligo_024)	-0.33	-0.15	-0.18	-0.23	0.07	-0.05	0.02	-0.49	-0.41	-0.48	-0.37	0.11	-0.17	0.70	0.60	1.00	0.91	0.11	-0.41	-0.16	-0.29	-0.20	-0.27	-0.36	-0.42	0.08	-0.27	-0.24	-0.06	-0.10
H. parainfluenzae 99.2%	-0.40	-0.33	-0.29	-0.31	-0.09	-0.23	-0.14	-0.52	-0.48	-0.41	-0.36	0.14	-0.15	0.79	0.58	0.91	1.00	0.11	-0.37	-0.11	-0.18	-0.12	-0.17	-0.22	-0.30	0.23	-0.21	-0.17	0.05	-0.01
F. periodonticum	-0.47	-0.29	-0.31	-0.03	-0.11	-0.24	-0.20	-0.26	-0.50	-0.25	-0.03	0.11	-0.28	0.01	0.36	0.11	0.11	1.00	0.30	0.60	0.43	0.09	0.08	0.05	-0.04	-0.06	-0.26	0.02	-0.08	-0.11
C. concisus 99.2%	-0.19	-0.06	-0.14	0.06	-0.20	-0.19	-0.17	0.21	-0.10	0.22	0.43	0.07	0.02	-0.28	-0.16	-0.41	-0.37	0.30	1.00	0.40	0.57	0.04	0.08	0.35	0.34	0.03	0.09	-0.01	-0.07	-0.12
L. umeaense	-0.37	-0.27	-0.31	-0.07	-0.22	-0.31	-0.23	-0.02	-0.25	0.09	0.19	0.05	-0.15	-0.15	0.11	-0.16	-0.11	0.60	0.40	1.00	0.49	0.24	0.25	0.29	0.17	0.07	0.02	-0.03	0.00	0.01
O. sp. 96.9%	-0.32	-0.32	-0.31	-0.15	-0.39	-0.40	-0.37	-0.12	-0.34	0.12	0.07	0.12	-0.06	-0.18	0.07	-0.29	-0.18	0.43	0.57	0.49	1.00	0.28	0.31	0.61	0.51	0.28	0.22	0.27	0.08	0.15
V. parvula	-0.30	-0.42	-0.34	-0.04	-0.29	-0.23	-0.22	0.03	0.04	0.36	0.10	0.06	-0.21	-0.15	-0.09	-0.20	-0.12	0.09	0.04	0.24	0.28	1.00	0.97	0.40	0.33	0.20	0.23	0.39	0.19	0.22
V. parvula 99.2%	-0.30	-0.42	-0.36	-0.04	-0.33	-0.28	-0.25	0.10	0.07	0.47	0.14	0.06	-0.18	-0.20	-0.12	-0.27	-0.17	0.08	0.08	0.25	0.31	0.97	1.00	0.43	0.37	0.22	0.23	0.42	0.19	0.20
S. parasanguinis	-0.22	-0.38	-0.35	-0.28	-0.52	-0.47	-0.46	-0.03	-0.20	0.35	0.09	0.08	-0.07	-0.25	0.02	-0.36	-0.22	0.05	0.35	0.29	0.61	0.40	0.43	1.00	0.77	0.54	0.67	0.50	0.44	0.57
S. salivarius/vestibularis	-0.17	-0.28	-0.25	-0.13	-0.42	-0.37	-0.33	0.07	-0.07	0.45	0.19	0.02	-0.01	-0.30	-0.16	-0.42	-0.30	-0.04	0.34	0.17	0.51	0.33	0.37	0.77	1.00	0.37	0.49	0.38	0.25	0.37
R. aeria 95.4%	-0.26	-0.41	-0.39	-0.43	-0.47	-0.52	-0.49	-0.30	-0.38	0.10	-0.11	0.10	-0.06	0.25	0.26	0.08	0.23	-0.06	0.03	0.07	0.28	0.20	0.22	0.54	0.37	1.00	0.32	0.37	0.45	0.44
S. cristatus/gordonii/sinensis	0.18	-0.12	-0.10	-0.26	-0.39	-0.25	-0.32	0.02	0.08	0.25	0.00	-0.03	0.09	-0.30	-0.13	-0.27	-0.21	-0.26	0.09	0.02	0.22	0.23	0.23	0.67	0.49	0.32	1.00	0.39	0.39	0.61
G. sanguinis	0.00	-0.23	-0.21	-0.22	-0.37	-0.35	-0.36	-0.14	-0.08	0.21	-0.11	0.00	-0.05	-0.20	0.02	-0.24	-0.17	0.02	-0.01	-0.03	0.27	0.39	0.42	0.50	0.38	0.37	0.39	1.00	0.41	0.54
G. adjacens	-0.06	-0.39	-0.34	-0.26	-0.33	-0.34	-0.33	-0.15	-0.17	0.14	-0.05	-0.10	-0.07	0.04	0.17	-0.06	0.05	-0.08	-0.07	0.00	0.08	0.19	0.19	0.44	0.25	0.45	0.39	0.41	1.00	0.63
S. peroris	0.05	-0.28	-0.27	-0.32	-0.39	-0.34	-0.35	-0.24	-0.19	0.03	-0.17	-0.16	-0.11	-0.05	0.14	-0.10	-0.01	-0.11	-0.12	0.01	0.15	0.22	0.20	0.57	0.37	0.44	0.61	0.54	0.63	1.00

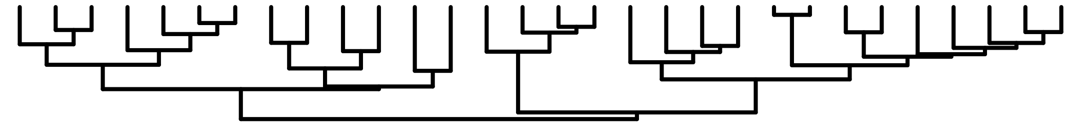

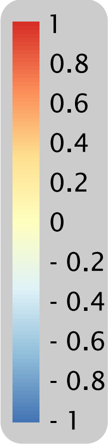

Figure S1. Time series correlation heat map. The 30 most abundant oligotypes in the time series data, measured by total reads, are shown. Pearson correlation coefficients were calculated for each pair of oligotypes for the read counts measured in the male subject in days 66-420. Heat map is a visual representation of the correlation coefficients; the coefficients themselves are shown in each square.
